# Repeat surgery of recurrent glioma for molecularly informed treatment in the age of precision oncology: A risk–benefit analysis

**DOI:** 10.1007/s11060-024-04595-5

**Published:** 2024-02-09

**Authors:** Obada T. Alhalabi, Philip Dao Trong, Manuel Kaes, Martin Jakobs, Tobias Kessler, Hannah Oehler, Laila König, Tanja Eichkorn, Felix Sahm, Jürgen Debus, Andreas von Deimling, Wolfgang Wick, Antje Wick, Sandro M. Krieg, Andreas W. Unterberg, Christine Jungk

**Affiliations:** 1 Department of Neurosurgery, Heidelberg University Hospital, Im Neuenheimer Feld 400, 69120 Heidelberg, Germany; 2 Department of Neurosurgery, Medical Faculty, Heidelberg University, Heidelberg, Germany; 3 Department of Neurosurgery, Division for Stereotactic Neurosurgery, University Hospital Heidelberg, Heidelberg, Germany; 4 Clinical Cooperation Unit Neurooncology, German Cancer Research Center (DKFZ), Heidelberg, Germany; 5 Department of Neurology and Neurooncology Program, National Center for Tumor Diseases, University Hospital Heidelberg, Heidelberg, Germany; 6 Department of Radiation Oncology, Heidelberg Ion Beam Therapy Centre (HIT), National Center for Radiation Oncology (NCRO), Heidelberg Institute for Radiation Oncology (HIRO), Heidelberg University Hospital, Heidelberg, Germany; 7 Department of Neuropathology, University Hospital Heidelberg, Heidelberg, Germany; 8 CCU Neuropathology, German Consortium for Translational Cancer Research (DKTK), German Cancer Research Center (DKFZ), Heidelberg, Germany

**Keywords:** Recurrent glioma, Molecular diagnostics, Targeted therapy, Precision oncology, Surgical complications

## Abstract

**Purpose:**

Surgery for recurrent glioma provides cytoreduction and tissue for molecularly informed treatment. With mostly heavily pretreated patients involved, it is unclear whether the benefits of repeat surgery outweigh its potential risks.

**Methods:**

Patients receiving surgery for recurrent glioma WHO grade 2–4 with the goal of tissue sampling for targeted therapies were analyzed retrospectively. Complication rates (surgical, neurological) were compared to our institutional glioma surgery cohort. Tissue molecular diagnostic yield, targeted therapies and post-surgical survival rates were analyzed.

**Results:**

Between 2017 and 2022, tumor board recommendation for targeted therapy through molecular diagnostics was made for 180 patients. Of these, 70 patients (38%) underwent repeat surgery. IDH-wildtype glioblastoma was diagnosed in 48 patients (69%), followed by IDH-mutant astrocytoma (*n* = 13; 19%) and oligodendroglioma (*n* = 9; 13%). Gross total resection (GTR) was achieved in 50 patients (71%). Tissue was processed for next-generation sequencing in 64 cases (91%), and for DNA methylation analysis in 58 cases (83%), while immunohistochemistry for mTOR phosphorylation was performed in 24 cases (34%). Targeted therapy was recommended in 35 (50%) and commenced in 21 (30%) cases. Postoperatively, 7 patients (11%) required revision surgery, compared to 7% (*p* = 0.519) and 6% (*p* = 0.359) of our reference cohorts of patients undergoing first and second craniotomy, respectively. Non-resolving neurological deterioration was documented in 6 cases (10% vs. 8%, *p* = 0.612, after first and 4%, *p* = 0.519, after second craniotomy). Median survival after repeat surgery was 399 days in all patients and 348 days in GBM patients after repeat GTR.

**Conclusion:**

Surgery for recurrent glioma provides relevant molecular diagnostic information with a direct consequence for targeted therapy under a reasonable risk of postoperative complications. With satisfactory postoperative survival it can therefore complement a multi-modal glioma therapy approach.

**Supplementary Information:**

The online version contains supplementary material available at 10.1007/s11060-024-04595-5.

## Introduction

Glial tumors make up about 70% of primary intracranial tumors [1]. The most aggressive, glioblastoma (GBM), isocitrate dehydrogenase (IDH) wildtype, is a therapy-resistant systemic brain disease with dismal prognosis [2, 3]. Despite multimodal treatment including maximal safe resection, radio- and chemotherapy, recurrence of diffuse glioma is almost inevitable. Many trials on systemic therapy for recurrent glioma have failed in the past [4, 5]. Latest data indicate a median overall survival of 15 months for patients with GBM, isocitrate dehydrogenase (IDH) wildtype [6]. Even IDH-mutant diffuse gliomas of lower WHO grades, albeit showing longer survival rates, almost always involve a long path of multimodal salvage therapies [7, 8].

Over the past years, genomic studies have revolutionized our understanding of the biology, diagnosis and classification of intracranial tumors. The 5th edition of the WHO classification of tumors of the central nervous system (CNS) has combined molecular and histopathological features to provide integrated diagnoses of CNS tumors [9]. Indeed, current high-throughput sequencing pipelines do not only increase the reliability of current tumor diagnoses but also provide a workup for a more personalized approach in neurooncological therapy [10–12].

However, most salvage therapies do not account for possible post-therapeutic changes in tumor biology especially in IDH*-*mutant glioma and show limited treatment efficacy and inevitable further tumor progression [13–17]. Therefore, sampling of current tumor tissue at progression to inform on relevant molecular alterations for targeted therapy has hence become an increasingly relevant modality of last-line therapy [18].

At the same time, repeat surgery has been established as an option for amenable patients with recurrent glioma, with multiple studies on a viable post-surgery survival benefit of patients with GBM and low-grade glioma evolving in the past [19–23]. This is especially true for patients with a high extent of resection, under a reasonable rate of permanent neurological deficits, ranging from 4 to 8%, and surgical complication rates of 9 to 22% [24–29].

In this regard, surgery for recurrent glioma could provide, in addition to cytoreduction, tissue that recapitulates the real-time post-therapeutic tumor biology to help provide further targeted therapy options at tumor recurrence. However, with mostly heavily pretreated patients involved, little is known as to whether benefits of repeat surgery aiming at molecularly informed treatment outweigh its potential surgical and neurological complications.

## Methods

In this retrospective study, we analyzed clinical, histopathological, molecular, surgical and follow-up data of 180 patients evaluated by the interdisciplinary tumor board of our center with the recommendation of tissue sampling of recurrent intracranial tumors for molecularly informed personalized treatment. Only patients undergoing repeat surgery for diffuse glioma (gross total resection (GTR), subtotal resection (STR), open biopsy or stereotactic biopsy) at our center between 2017 and 2022 were included (*n* = 70). In resection cases, the extent of resection (GTR or STR) was evaluated on early postoperative MRIs (within 48 h), with no residual contrast-enhancing (in case of GBM) or FLAIR (in case of IDH-mutant gliomas FLAIR-hyperintense FLAIR-hyperintense) tumor volume defined as GTR.. Cases with repeat surgery at other centers and cases in which repeat surgery did not take place were excluded (*n* = 102). In addition, a reference cohort of patients undergoing surgery for newly diagnosed diffuse glioma = ‘primary reference cohort’ and recurrent gliomas = ‘recurrent reference cohort’ (each as a separate group) treated at our center in 2022 was used to compare complication rates of repeat surgery with primary surgery for intracranial gliomas. This reference data is available under [30].

### Statistical analysis

Patient characteristics were analyzed using descriptive statistics. Continuous variables are reported as mean ± standard deviation or median (and interquartile range (IQR)). Ordinal and nominal variables are presented as numbers and percentages. Missing data are designated as such. Comparison of nominal variables between groups was performed using Fisher’s exact test. Survival analysis was performed using the Gehan-Breslow-Wilcoxon test and the Log-rank (Mantel-Cox) test. All statistical analyses were performed using Graphpad PRISM (Version 9).

## Results

### Repeat surgery of recurrent glioma for molecularly informed treatment

**Fig. 1:**
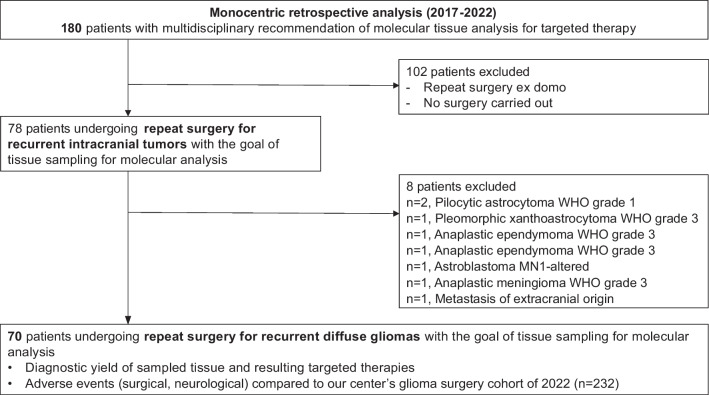
Study flow-chart. WHO = World Health Organization

Between 2017 and 2022, the multidisciplinary tumor board at our center recommended analysis of current tumor tissue for targeted therapy in 180 patients with recurrent intracranial tumors. Of those, 102 patients (57%) were excluded from the analysis, either because repeat surgery took place at a different institution or because patients did not undergo repeat surgery at all, e.g. because the patient restrained from it or because molecular diagnostics was performed on tumor tissue derived from previous surgery. Overall, there was no significant difference in the clinical condition of patients not undergoing surgery at our institution as informed by their Karnofsky Performance Status (KPS) compared to the analyzed cohort (82.3% vs 86.1%, *p* = 0.06, two-tailed t-test). However, males were overrepresented in the cohort of excluded patients (68% vs. 44% in the study cohort, *p* = 0.002, Chi-square test). In addition, a higher proportion of deeply located lesions (basal ganglia and brain stem) with a significantly higher rate of white matter involvement was noted in the group not undergoing surgery at our institution (21% vs. 6% in the study cohort, *p* = 0.03, Chi-square test, Supplementary Table 1). Because we focused our further analysis on diffuse gliomas, 8 additional cases (two cases of pilocytic astrocytoma WHO grade 1, one case of each: pleomorphic xanthoastrocytoma, WHO grade 3; anaplastic meningioma WHO grade 3; anaplastic ependymoma WHO grade 3; Astroblastoma, MN1 altered; metastasis of extracranial origin and pituitary adenocarcinoma) were excluded (Fig. 1).

**Fig. 2:**
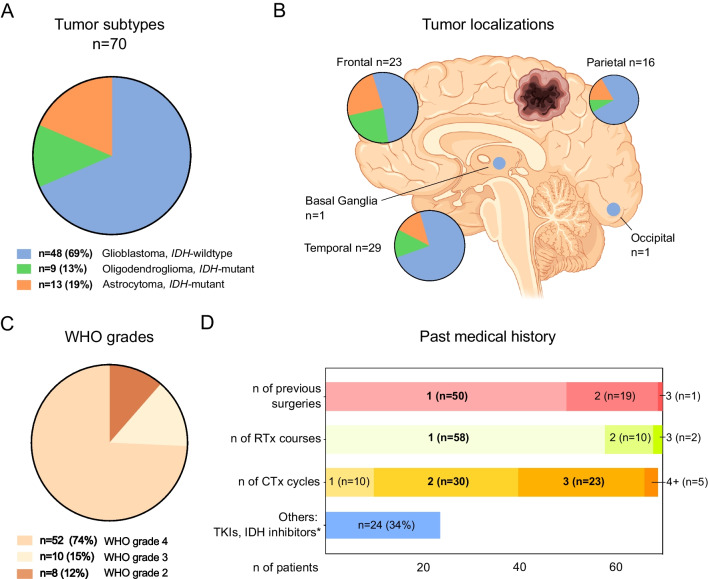
Patient and tumor characteristics. **A**: Tumor subtypes, IDH = isocitrate dehydrogenase. **B**: Tumor (predominant) localizations, color coding according to legend (A) Figure created in part using biorender.com. **C**: Tumor WHO grades. **D**: Past medical history. RTx = Radiotherapy, CTx = Chemotherapy, TKI = Tyrosine kinase inhibitor, *Includes Bevacizumab (*n* = 6), tumor peptide vaccinations (*n* = 6), IDH-inhibitors (*n* = 2)

All 70 remaining cases were diagnosed as diffuse gliomas: 48 cases of WHO grade 4 IDH-wildtype glioblastoma, including three cases of gliosarcoma), 13 cases of IDH-mutant astrocytoma WHO grade 2, 3, and 4, and 9 cases of IDH-mutant oligodendroglioma, WHO grade 2 and 3. About one third of the diagnosed cases (*n* = 22) included tumors harboring *IDH* mutations. Demographically, this patient cohort included 39 females and 31 males with a median age of 54 years (interquartile range, IQR of 49 to 63). Tumors were mostly localized within the frontal (33%) and temporal (41%) lobes, (Fig. 2). Most patients showed a similar past medical history. The “average” patient included in the analysis had undergone one surgical procedure for tumor resection (*n* = 50, 71%), with further 27% of patients showing a history of two previous craniotomies for tumor resection. In most of the cases, one course of radiotherapy (*n* = 58, 83%) and two (*n* = 30, 46%) to three (*n* = 23, 39%) courses of chemotherapy had taken place prior to repeat surgery. Most patients had received primarily temozolomide as adjuvant chemotherapy (64 patients, 91%). 24 patients (34%) had also received further therapies prior to repeated surgery, including anti-angiogenic drugs, tumor neo-antigen vaccines and small molecular inhibitors, like IDH-inhibitors (Table 1, Fig. 2).

**Table 1:** Patient characteristics

Patient Characteristic	*n* = 70
Age at repeat surgery (years)	
Median, (IQR)	54.0 (13.9)
Mean, (SD)	53.8 (11.3)
Gender	
Male, (%)	31 (44%)
Female, (%)	39 (56%)
Molecular Diagnoses	
Glioblastoma, IDH-wildtype, WHO grade 4, (%)	48 (73%)
Diffuse astrocytoma, IDH-mutant, (%)	13 (19%)
WHO grade 2	6
WHO grade 3	3
WHO grade 4	4
Oligodendroglioma, IDH-mutant, (%)	9 (13%)
WHO grade 2	2
WHO grade 3	7
Tumor localizations, (%)	
Frontal	23 (33%)
Parietal	16 (23%)
Temporal	29 (41%)
Occipital	1 (1%)
Basal ganglia	1 (1%)
Tumor side, (%)	
Right	35 (50%)
Left	35 (50%)
N of previous surgeries	
1	50 (71%)
2	19 (27%)
3	1 (2%)
N of previous chemotherapy cycles	
None	2 (3%)
1	10 (14%)
2	30 (43%)
3	23 (33%)
4 or more	5 (6%)
Applied agents	
TMZ (temozolomide)	64 (91%)
CCNU (lomustine)/ VP-16 (etoposid)	48 (69%)
BCNU (carmustine)	1 (1%)
PCV (procarbazine, lomustine and vincristine)	1 (1%)
Other modalities	
Bevacizumab	6 (9%)
IDH-inhibitors	2 (3%)
Tumor-treating fields	6 (9%)
Tumor vaccines	2 (3%)
Other*	6 (9%)
N of previous radiotherapy courses	
1	58 (83%)
2	10 (14%)
3	2 (3%)

*IQR* Inter-quartile range, *SD* Standard deviation, *IDH* isocitrate dehydrogenase, *WHO* World Health Organization. *Therapy agents include Chloroquine, Irinotecan, Palbociclib, Atezolizumab

### Diagnostic yield of repeatsurgery and its implication for targeted therapies

**Fig. 3:**
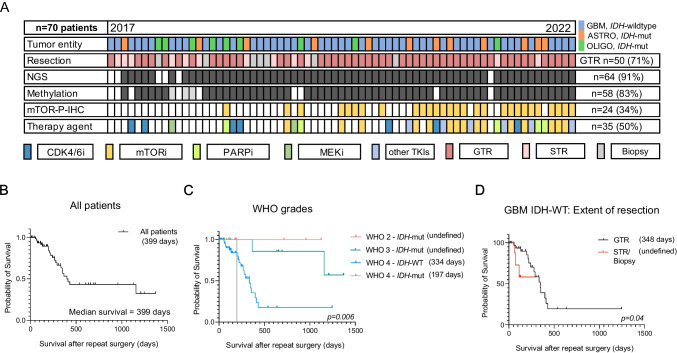
Oncoplot of available tissue data with overall survival (OS) after surgery in different patient groups in the patient cohort. **A**: Consecutive patients are listed horizontally in chronological order (time of surgery from 2017 to 2022). NGS = Next-generation sequencing. mTOR-P-IHC = Phospho-mTOR Immunohistochemistry. IDH = isocitrate dehydrogenase. CDK4/6i = Cycline-dependent Kinase 4/6 inhibitors. mTORi = mammalian target of rapamycin inhibitor. PARPi = Poly (ADP-ribose) polymerase inhibitor. MEKi = Mitogen-activated protein kinase kinase inhibitor. GTR = Gross Total Resection, STR = Subtotal resection **B**: All patients in the cohort (median survival 399 days). **C**: Survival after surgery based on WHO classification grades (WHO 2: undefined, WHO 3: undefined, WHO 4, isocitrate dehydrogenase wild type, IDH-WT: 334 days, WHO 4, IDH-mut: 197 days. **D**: Survival in GBM, IDH-WT patients after gross total resection (GTR, 348 days) vs all other surgical modalities (subtotal resection – STR and biopsies, survival undefined) in this cohort, *p* = 0.04, Gehan-Breslow-Wilcoxon test

In 66 cases (94%), tissue samples from repeat surgery were further processed for next-generation sequencing (NGS, performed in *n* = 64, 91% of the cases) and DNA methylation profiling (performed in *n* = 58, 83% of the cases), with methylation data from previous tissue used in 3 further cases). In four cases (6%), further molecular workup was omitted, either because radiation necrosis instead of vital tumor tissue was diagnosed (*n* = 1) or because the postoperative condition of the patients deteriorated rapidly, rendering them non-amenable for further therapies (*n* = 3). Immunohistochemistry for mTOR phosphorylation (mTOR-P-IHC) was carried out in 34% of the cases (*n* = 24). A statistically non-significant difference was noted in the proportion of patients with actionable targets in patients undergoing biopsies (2/9, 22%) compared to patients undergoing gross total and subtotal resection (33/61 cases, 54%, *p* = 0.151, Fisher’s exact test). In 35 cases (50%), a recommendation for targeted therapy was made based on tissue analyses. Recommended agents included mTOR inhibitors (*n* = 15), CDK 4/6 and PARP inhibitors (*n* = 6 each), MEK inhibitors (*n* = 3), and further tyrosine kinase inhibitors (TKIs, *n* = 5). Targeted therapy was commenced in 21 cases (30% of the complete cohort). In the remaining 14 cases (20%) patients either deceased or their general condition deteriorated before targeted therapy could be initiated. This included 8 cases where patients were lost to follow up and further 6 cases of patients that succumbed to tumor progress while applications for health insurance approval were either pending or refused (Fig. 3A).

Next, we examined the post-surgery survival of the patients of this cohort. At the time of this analysis, 44 of 70 patients were censored. Across all tumor types and WHO grades, the median survival after repeat surgery was 399 days (Fig. 3B), indicating a median survival long enough for patients to benefit from the personalized targeted therapies that were determined based on the sampled tumor tissue. As expected, patients with WHO grade 4 tumors showed the worst survival after surgery, compared to WHO grade 2 and grade 3 tumors (348 days in WHO grade 4 vs. undefined in other groups, *p* = 0.03, Gehan-Breslow-Wilcoxon test, Fig. 3C). Indeed, patients with IDH-wildtype GBM conferred a post-surgery survival rate of 334 days. To confirm the prognostic benefit of GTR in the context of this study, we compared survival after repeat surgery in GBM patients undergoing GTR (348 days, *n* = 34) to that of all other GBM patients (undergoing STR, open or stereotactic biopsies), with undefined survival due to small sample size in the latter (*n* = 10, *p* = 0.04, Gehan-Breslow-Wilcoxon test, Fig. 3D).

### Surgical and neurological risk after repeat surgery: A comparative analysis

In this cohort, GTR was possible in 50 cases (71%). Other surgical modalities included subtotal resection (*n* = 11, 16%), and open or stereotactic biopsies (*n* = 9, 13%). This is in line with the number of cases where tissue sampling was the primary goal of surgery (*n* = 9, see Table 2). To establish a workable frame for a risk–benefit analysis in this cohort, we compared the rate of postoperative surgical complications and neurological deterioration with that of all other patients undergoing surgery for newly diagnosed or first glioma recurrence at our center in 2022 (*n* = 232). This ‘reference cohort’ was further divided into patients with repeat surgery on first tumor recurrence, i. e. patients receiving their second craniotomy. (*n* = 50, recurrent reference cohort) and patients receiving surgery (first craniotomy) for newly diagnosed diffuse glioma (*n* = 182, primary reference cohort).

**Table 2:** Surgical and neurological outcomes

Outcome	Repeat surgery cohort *n* = 70 (%)	Recurrent reference cohort *n* = 50 (%)	*p*-Value*	Primary reference cohort *n* = 182 (%)	*p*-Value*
Extent of resection			0.02		0.002
Gross total resection (GTR)**	50 (71)	27 (54)		77 (42)	
Subtotal resection (STR)**	11 (16)	19 (38)		57 (31)	
Biopsy	9 (13)	4 (8)		48 (26)	
Previous intent of mere tissue sampling	9 (13)				
Complications requiring surgical intervention	7 (11)	3 (6)	0.519	12 (7)	0.359
Wound healing disorder without CSF fistula	3 (4)	2 (4)		4 (2)	
CSF fistula	2 (3)	0 (0)		2 (1)	
CSF circulation disorder	2 (3)	0 (0)		1 (< 1)	
Postoperative intracranial hemorrhage	0 (0)	1 (2)		5 (3)	
Postoperative neurological deterioration	13 (19)	6 (12)	0.448	23 (13)	0.233
Hemiparesis	6 (9)	2 (4)		5 (3)	
Aphasia	4 (7)	2 (4)		11 (6)	
Hemianopsia	1 (1)	1 (2)		3 (2)	
Aggravation of focal seizures	2 (3)	1 (2)		2 (1)	
Other	0 (0)	0 (0)		2 (1)	
Non-resolving neurological deterioration	7 (10)	3 (4)	0.519	14 (8)	0.612

Please note that all cases of aggravated focal seizures were transient. CSF = cerebrospinal fluid. SDH = subdural hematoma. *Fisher’s exact test. **verified through early postoperative MRI as a nodular contrast enhancing/ FLAIR-hyperintense residual tumor (within 48 h). Data from recurrent reference and primary reference cohort available under [30]

The surgical revision rate in the current study cohort (repeat surgery cohort) was 11% (*n* = 7), compared to 7% and 6% in the recurrent and primary reference cohorts, respectively (*p* = 0.519 and *p* = 0.359, Fisher's exact test). Complications requiring revision surgery in the repeat surgery study cohort included superficial wound healing disorders (*n* = 3), CSF (cerebrospinal fluid) fistula (*n* = 2) or CSF circulation disorders requiring placement of a drain into the resection cavity (*n* = 2). No postoperative hematomas were observed in the repeat surgery study cohort. We next examined the role of repeat surgery on newly developed or aggravated neurological deficits. Postoperative neurological deterioration mainly included hemiparesis and aphasia (*n* = 6 and *n* = 4, respectively). In two patients, an aggravation of focal seizure frequency and in one case a postoperative hemianopsia were noted, setting the rate of immediate postoperative neurological deterioration to 19% (13/70 cases), with no statistically significant differences to the recurrent and primary ‘therapy-naïve’ reference cohorts, which showed postoperative neurological deterioration rates of 12% and 13% (*p* = 0.448 and 0.233, respectively, Fisher’s exact test). Importantly, in all cohorts, deficits resolved in almost half of the patients, with non-significant differences in permanent deficits between patients of the repeat surgery (10%) vs. 4% and 8% in patients of the reference cohorts, *p* = 0.612, Fisher’s exact test, Table 2).

## Discussion

In the current analysis, 50% of all patients undergoing repeat surgery after a tumor board recommendation for molecularly informed treatment emerged with a recommendation for targeted therapy. In 40% of these patients, therapy could not be initiated for reasons not related to the surgical procedure itself but because of rapid tumor progression while health insurance approval for therapy was pending, impeding commencement of treatment. Ultimately, one third of all patients undergoing repeat surgery received targeted therapies. The slightly higher risk for perioperative complications and transient neurological deterioration compared to our institutional reference cohort (including both recurrent and therapy-naïve, newly diagnosed glioma patients) was non-significant. Similarly, differences in rates of post-surgical permanent neurological deficits were also non-significant.

Although tumor tissue sampling was the primary intention of surgical intervention in this cohort, it was usually not the mere achievement: GTR was possible in 70% of the cases. In our study, patients with IDH-wildtype GBM conferred a median survival after repeat surgery of 348 days (11.6 months) after GTR. This is comparable to survival rates reported in previous trials focusing on GBM patients receiving their first re-resection, which range between 11.9 and 12.9 months [20, 29]. It is important to note that, due to the small sample size and heterogenous treatment regimens, a direct prognostic benefit from the choice of targeted therapy after surgery cannot be deduced in this cohort.

In the light of recent advances in molecular diagnostics and their potential to deliver personalized therapeutic strategies this study aimed to highlight a further dimension of the benefits of repeat surgery: The potential of tissue sampling to inform on therapeutic targets under a reasonable perioperative risk of surgical complications and neurological deterioration. Procedures like STR or even biopsy were still able to inform on therapeutic targets according to our findings, rendering them amenable intervention options in individualized settings when GTR is not viable. Nevertheless, given the high proportion of patients undergoing GTR in this cohort, our data indicate that even in the context of tissue sampling for molecularly informed treatment as a primary intent, it is worth evaluating the possibility of a maximized re-resection when weighed out against a ‘mere’ biopsy.

In this cohort, the diagnostic yield for actionable targets was 55% in resection cases and 22% in biopsy cases (difference non-significant, primarily limited by the small sample size in the biopsy group). The overall rate of actionable targets in this current cohort was 50%, which is comparable to what is found in reports identifying targetable mutations in recurrent glioma patients. To our knowledge, only one comparable analysis has been described in the literature. In a study by Blobner et al. [31] the rate of actionable targets identified in glioma patients was 69%, but also included *IDH* mutations (30%), which was regarded as pre-known in our analysis, making the rate of ‘novel’ mutations similar in both studies (at about 40% to 50%). Of note, in the study by Blobner et al., only 17% of all patients (*n* = 72) could commence targeted therapy, which is slightly lower than our study (30%). Beyond the molecular work-up including methylome profiling and panel sequencing of tumor material, Phospho-mTOR-IHC was applied on tumor samples to detect mTOR activation. Because phosphorylations are dynamic post-translational modifications that could point to adaptive resistance mechanisms of current therapy [32, 33], sampling of tumors at their recurrence could therefore also provide an opportunity to inform on such mechanisms, as shown in this analysis. Most likely, given the advancements in molecular diagnostics and our increasing understanding of glioma biology, the therapeutic yield will naturally increase beyond 50% in the coming years.

Given the intractable situation of a recurrent glioma without further standard treatment options, patients in this cohort show a considerable post-surgical median survival of about 13 months and about 12 months for GBM patients. This postoperative survival was, according to the data we present, long enough for patients to receive targeted therapy. It has to be noted, however, that 40% of patients with a treatment target identified had deceased before treatment could be commenced, amongst others because of rapid postoperative tumor progression. This observation emphasizes the importance of patient selection.

The notion that patients with recurrent glioma are at a higher risk of surgical and neurological complications [34] because of intensive pre-treatment, especially in high-grade gliomas, necessitates weighing out potential benefits of such individualized treatment approaches against the presumed risks of surgical intervention. In this cohort, the surgical complication rate (11%) did not significantly differ from rates of therapy-naïve patients with newly diagnosed gliomas in our primary reference cohort (6%) or glioma patients undergoing repeat surgery in our recurrent reference cohort (7%). Moreover, the surgical complication rate we describe is comparable to what has been reported in the literature for similar patient cohorts (surgical complication rates of up to between 8 to 30%) [29, 35, 36]. It is of note that the surgical complications observed in this analysis belonged to the less severe spectrum (Clavien-Dindo classification ≤ 3), and did not involve prolonged intensive care unit stays or death [37].

Permanent neurological deficits were observed in 10% of the study cohort, which is slightly higher than in the reference cohort of newly diagnosed glioma patients (4%, differences statistically non-significant). This effect could be attributed to the higher GTR (71%) rate in this cohort, compared to newly diagnosed glioma patients (42%). The observation is comparable to previous studies on recurrent glioma, citing neurological deterioration rates of 8% to 20% [29, 38–40].

Care should be taken when interpreting the data presented due to several limitations, especially with regards to postoperative survival rates. Owing to this individualized approach and the retrospective study design, the patient cohort was heterogeneous in terms of tumor subtypes, WHO grades and targeted therapies, each of them with a potential prognostic impact. For instance, only 9 patients with IDH-mutant oligodendroglioma and 13 patients with IDH-mutant astrocytoma were included, and even in the largest subgroup of IDH-wildtype GBM patients, the variety of surgical approaches, tumor localizations and targeted therapies applied does not provide data with enough ground to reliably assess the efficacy of targeted therapy on patient survival, which is also beyond the intended scope of this analysis. When examining survival rates, it should be noted that a high number of patients was censored, mostly due to loss of follow up. However, it is safe to assume that a certain proportion of these patients, especially with IDH-mutant WHO 2 and 3 gliomas (these WHO grades have the highest proportion of censored patients in this analysis), had not yet been deceased by the time of this analysis. Still, general observations on survival rates in this cohort could still be made and do confirm previous reports [29].

From a surgical point of view, this analysis did not investigate specific tumor locations pertaining to, for example, eloquence, which would deem the perioperative risk for neurological deterioration higher than in non-eloquent locations [41]. Also, as known from previous studies, volumetric analysis of residual tumor instead of qualitative assessment of GTR vs STR could have helped stratify patients with regards to the effect of the absolute residual tumor volume on survival [42]. This study also does not examine the effect of these interventions on the quality of life or the cognitive performance of affected patients, especially in patients with lower-grade tumors expected to survive longer with a potentially higher burden of disease [43, 44]. From the patients’ point of view, the question as to whether the risks taken and efforts spent under this individualized approach are justified may not be potentially answered by the duration but rather by the quality of prolonged survival. In addition, the aspect of progressive disease itself causing neurological deterioration is not addressed by our data. Here, a control group of patients receiving targeted therapies without previous surgery for tissue sampling may help address this confounding factor. We also acknowledge that this is a highly advanced and individualized approach that is not readily available in other regions or healthcare systems, and therefore has wide implications for the generalizability of this approach in an international setting and the standard of care that could be provided to these patients [45, 46].

Nevertheless, the data we present lays out that in the case of treatment-refractory recurrent glioma, surgical resection for tissue sampling bears a realistic potential of providing relevant therapeutic targets in addition to a survival-relevant cytoreduction, with a reasonable postoperative surgical and neurological complication rate. With a sizable proportion of patients also commencing personalized targeted therapy, this work helps involved neuro-oncologists and neurooncological surgeons weigh out the risks and benefits of surgery and provide patients, families and health-care providers with realistic expectations when offering such surgical interventions in the future.

## Conclusion

Surgery for recurrent glioma aiming at molecularly informed treatment is associated with a reasonable surgical morbidity and an acceptable risk of neurological deficits that does not seem to be significantly higher than in primary surgery. With GTR achieved in most cases and druggable targets identified in about half of the patients, a targeted therapy could be part of a multimodal approach in patients with recurrent glioma. Further subgroup analyses with a larger patient cohort could help provide optimized patient stratification for predicting risks of peri-operative complications to aid tumor-board based decision making in this patient cohort.

### Supplementary Information

Below is the link to the electronic supplementary material.

Supplementary file1 (DOCX 18 KB)

## Data Availability

The datasets generated during and/or analyzed during the current study are available from the corresponding author on reasonable request.
