# Supplementary material for: Repeat surgery of recurrent glioma for molecularly informed treatment in the age of precision oncology: A risk–benefit analysis
Source: J Neurooncol. 2024 Feb 9;167(2):245–55. doi: 10.1007/s11060-024-04595-5 (PMC11023957; doi:10.1007/s11060-024-04595-5)
Supplement: Supplementary file 1 — Supplementary file1 (DOCX 18 KB) [file 11060_2024_4595_MOESM1_ESM.docx]

| **Variable** | **Surgery at institution (n=70)** | **No surgery at institution**  **(n=102)** | **p-Value** |
| --- | --- | --- | --- |
| Gender  Male (%)  Female (%) | 31 (44%) 39 (56%) | 69 (68%)  33 (32%) | 0.002* |
| Baseline KPS (%) | 86,1 | 82,3 | 0.06** |
| Tumor localizations, (%)  Frontal  Parietal  Temporal  Occipital  Basal ganglia  Brain stem  Cerebellum  Multifocal | 23 (33%) 16 (23%) 29 (41%) 1 (1%) 1 (1%)  0 (0%)  0 (0%)  0 (0%) | 30 (29%)  24 (24%)  22 (22%)  5 (5%)  13 (13%)  4 (4%)  1 (1%)  3 (3%) | 0.0004* |
| White matter Involvement (%) | 6 (9%) | 21 (21%) | 0.03* |

**Supplementary Table 1: Baseline characteristics of patients undergoing surgery compared with patients not undergoing surgery after tumor board recommendation. KPS = Karnofsy Performance Score.** *Chi-Square test, **two-tailed t-test.
